# Bispecific antibodies targeting BCMA, GPRC5D, and FcRH5 for multiple myeloma therapy: latest updates from ASCO 2023 Annual Meeting

**DOI:** 10.1186/s13045-023-01489-3

**Published:** 2023-08-03

**Authors:** Juanjuan Zhao, Quan Ren, Xinyuan Liu, Xiangqian Guo, Yongping Song

**Affiliations:** 1 Department of Hematology, The First Affiliated Hospital of Zhengzhou University, Zhengzhou, 450052 Henan China; 2 Institute of Biomedical Informatics, Bioinformatics Center, Henan Provincial Engineering Center for Tumor Molecular Medicine, School of Basic Medical Sciences, Henan University, Kaifeng, 475004 Henan China

**Keywords:** Multiple myeloma, Bispecific antibody, BCMA, GPRC5D, FcRH5

## Abstract

Several bispecific antibodies (bsAbs) targeting BCMA, GPRC5D, and FcRH5 are in clinical trials for heavily pretreated multiple myeloma (MM) patients. Teclistamab was approved for relapsed/refractory MM therapy in 2022, while elranatamab, linvoseltamab, F182112, talquetamab, and cevostamab are currently undergoing clinical trials. This study summarizes several latest reports on bsAbs for the treatment of MM from the ASCO 2023 Annual Meeting.

To the editor 

The first bispecific antibody (bsAb) for the treatment of relapsed/refractory multiple myeloma (RRMM), teclistamab, has been approved in 2022. Several bsAbs for MM are in clinical trials. We summarized the latest reports on bsAbs for MM therapy from the ASCO 2023 Annual Meeting (ASCO2023).

## Updates from clinical studies of BCMA × CD3 bsAbs

### Teclistamab

Teclistamab is the first BCMA × CD3 bsAb approved for the treatment of RRMM (Table 1). In the long-term follow-up (FU) from MajesTEC-1 study, 43% of RRMM patients (pts) achieved a complete response (CR) or stringent CR with teclistamab monotherapy (Table 2) [1]. Median disease-free survival (mPFS) and overall survival (mOS) were 12.5 months (m) and 21.9 m, respectively. Pts who achieved a partial response (PR) or better in phase 1, or a CR or better in phase 2, are eligible to transition from QW to Q2W. Cytokine release syndrome (CRS) and immune effector cell‐associated neurotoxicity syndrome (ICANS) occurred in 72% and 3% of pts, respectively, and 6 deaths related to treatment were reported. Grade 3/4 neutropenia and lymphopenia occurred in 65% and 33% of pts, respectively. A grade 3/4 infection occurred in 52% of pts.

**Table 1:** Properties of bispecific antibodies for multiple myeloma

Product	Target	Administration	Reference
Teclistamab	BCMA × CD3	SC	[1]
Elranatamab	BCMA × CD3	SC	[4]
Linvoseltamab	BCMA × CD3	IV	[7]
F182112	BCMA × CD3	IV	[8]
Talquetamab	GPRC5D × CD3	SC	[9]
Cevostamab	FcRH5 × CD3	IV	[13]

*Abbreviations: BCMA, B-cell maturation antigen; bsAbs, bispecific antibodies; FcRH5, Fc receptor-homolog 5; GPRC5D, G protein-coupled receptor family C group 5 member D; IV, intravenous; SC, subcutaneous*

**Table 2:** ASCO2023 updates from clinical studies of bispecific antibodies for multiple myeloma

Regimen	Teclistamab	Teclistamab	Elranatamab	Linvoseltamab	F182112	Talquetamab	Teclistamab + Talquetamab	Talquetamab + Daratumumab
Study	MajesTEC-1	NA	MagnetisMM-3	LINKER-MM1	NTP-F182112-001	MonumenTAL-1	RedirecTT-1	TRIMM-2
Disease	RRMM (without prior BCMA-directed therapies)	RRMM (with prior BCMA and GPRC5D-directed therapies)	RRMM (with and without prior BCMA-directed therapies)	RRMM (without prior BCMA-directed therapies)	RRMM (without prior BCMA-directed therapies)	RRMM (with and without prior T-cell-directed therapies)	RRMM	RRMM
Pts	165	24	123	252	16	288	63	65
mFU	22 m	1.3 m	12.8 m (range, 0.2–22.7)	2.3 m (200 mg); 4.7 m (50 mg)	3.1 m (range, 0.9–11.7)	14.9 m (QW); 8.6 m (Q2W); 11.8 m (prior T)	14.4 m (range, 0.5–21.9)	11.5 m (range, 1.0–27.3)
ORR	NA	60%	61% (objective response rate)	64% (200 mg); 50% (50 mg)	43.8% (95% CI, 19.8–70.1)	74% (QW); 73% (Q2W); 63% (prior T)	84%	78%
CR (sCR)	43%	NA	31.7%	NA	NA	NA	34%	45%
mDoR	24 m (95% CI, 16.2–NR)	NR	74.1% (95% CI, 60.5–83.6) at 12 m	NR	NA	NA	NR	NA
mPFS	12.5 m (95% CI, 8.8–17.2)	NR	57.1% (95% CI, 47.2–65.9) at 12 m	NA	NA	7.5 m (QW); 11.9 m (Q2W); 5.1 m (prior T)	NA	19.4 m
mOS	21.9 m (95% CI, 16.0–NR)	NR	62.0% (95% CI, 52.8–70.0) at 12 m	NA	NA	NA	NA	93% at 12 m
CRS	72%	41%	NA	37% (200 mg); 53% (50 mg)	81%	79% (QW); 75% (Q2W); 77% (prior T)	81%	78%
NAE/ICANS	ICANS: 3%	NAE: 13%	NA	ICANS ≥ G3: 2% (200 mg); 1% (50 mg)	NA	ICANS: 11% (QW); 11% (Q2W); 3% (prior T)	2%	5%
Clinical trial number	NCT03145181/ NCT04557098	NA (Retrospective study)	NCT04649359	NCT03761108	NCT04984434	NCT03399799/ NCT04634552	NCT04586426	NCT04108195
Reference	[1]	[2]	[4]	[7]	[8]	[10]	[11]	[12]

BCMA, B-cell maturation antigen; CR, complete response; CRS, cytokine release syndrome; G, grade; ICANS, immune effector cell‐associated neurotoxicity syndrome; m, months; mDoR, median duration of response; mFU, median follow-up; mPFS, median PFS; mOS, median OS; NA, not available; NAE, neurological adverse events; NR, not reached; ORR, overall response rate; OS, overall survival; PFS, progression-free survival; prior T, prior T-cell-directed therapies; Pts, patients; QW, quaque week; Q2W, quaque 2 weeks; RRMM, relapsed/refractory multiple myeloma; sCR, stringent CR;

A retrospective analysis of teclistamab in RRMM pts with prior BCMA and GPRC5D-directed therapies demonstrated an overall response rate (ORR) of 60% (9/15), with an ORR of 50% (5/10) among the subgroup receiving prior BCMA-targeted therapy at a mFU of 1.3 m (Table 2) [2]. CRS and neurotoxicity were observed in 41% and 13% of pts, respectively.

The MajesTEC-9 study is currently recruiting RRMM pts to compare the efficacy of teclistamab monotherapy versus (*vs*) pomalidomide + bortezomib + dexamethasone or carfilzomib + dexamethasone [3].

### Elranatamab

Elranatamab is a humanized bsAb targeting BCMA and CD3 (Table 1). In the MagnetisMM-3 study, elranatamab monotherapy was administered to 123 pts with RRMM (Table 2) [4]. The objective response and CR rates at a mFU of 12.8 m were 61% and 31.7%, respectively. The duration of response (DoR), PFS and OS at one year were 74.1%, 57.1%, and 62.0%, respectively. SC elranatamab was administered in a stepwise manner, with a target dose of 76 mg QW. Pts who received 6 cycles of treatment and achieved PR lasting more than 2 months were switched to 76 mg Q2W. Grade 3/4 infection included COVID-19 pneumonia (10.6%), pneumonia (7.3%), and sepsis (6.5%).

The MagnetisMM-6 study of elranatamab + daratumumab + lenalidomide *vs* daratumumab + lenalidomide + dexamethasone in newly diagnosed MM (NDMM) who are ineligible for transplant [5] and the MagnetisMM-7 study comparing elranatamab *vs* lenalidomide monotherapy in NDMM following autologous stem cell transplant (ASCT) are actively recruiting participants [6].

### Linvoseltamab

In the LINKER-MM1 study, linvoseltamab monotherapy was administrated to RRMM pts (Table 2) [7]. The ORR was 64% (*n* = 58) and 50% (*n* = 104) in the 200 mg and 50 mg cohorts, respectively, at a mFU of 2.3 m and 4.7 m. CRS occurred in 37% (200 mg cohort), and 53% (50 mg cohort) of the pts. ICANS ≥ grade 3 was reported in 2% (200 mg cohort) and 1% (50 mg cohort) of the pts. Infections ≥ grade 3 occurred in 26% (200 mg cohort) and 31% (50 mg cohort) of pts.

### F182112

F182112 was intravenously administrated to RRMM pts in a phase 1 study (Table 2) [8]. The ORR was 43.8% (7/16), with a mFU of 3.1 m. CRS remained the most common adverse event, occurring in 81% of pts. Grade 3/4 lymphopenia, neutropenia, and leukopenia occurred in 69%, 44%, and 50% of pts, respectively.

## Updates on bsAbs targeting GPRC5D or FcRH5 for MM

### Talquetamab

Talquetamab is a first-in-class G protein-coupled receptor family C group 5 member D (GPRC5D) × CD3 bsAb (Table 1) [9]. In the MonumenTAL-1 study, SC talquetamab was administrated to RRMM pts (Table 2) [10]. The mFU was 14.9 m, 8.6 m, and 11.8 m in cohorts 0.4 mg/kg QW, 0.8 mg/kg Q2W, and prior T-cell redirected therapies, with ORRs of 74%, 73%, and 63%, respectively. CRS and ICANS were observed in 79%, 75%, 77% and 11%, 11%, 3% of pts, respectively. The incidence of grade 3/4 infection was 22%, 16%, and 26%, respectively.

In the RedirecTT-1 study, talquetamab was used combined with teclistamab for RRMM treatment (Table 2) [11]. ORR was 84% and CR (including ≥ CR) was achieved in 34% of pts at a mFU of 14.4 m. CRS occurred in 81% of pts, and one pt experienced ICANS. Grade 3/4 neutropenia occurred in 75% of pts.

Talquetamab was used in combination with daratumumab for the treatment of RRMM in the TRIMM-2 study [12]. In the latest update, ORR and CR (≥ CR) rates were 78% and 45%, respectively, of the 65 pts included. OS rate at 12 m was 93% and mPFS was 19.4 m. The most common AE was CRS (78%), and 5% of pts experienced ICANS. Infections (≥ G3) occurred in 22% of pts.

### Cevostamab

Cevostamab, a bsAb targeting Fc receptor-homolog 5 (FcRH5) and CD3 (Table 1), is being evaluated in RRMM pts who have received prior anti-BCMA therapy, including anti-BCMA antibody-drug conjugates, CAR T cells, and anti-BCMA bsAbs [13]. Subject enrollment is currently in progress.

In summary, the majority of bsAbs for MM are directed toward BCMA, while the bsAbs targeting other antigens, GPRC5D, and FcRH5, are undergoing clinical trials. The approved teclistamab has demonstrated profound and enduring responses in RRMM pts naïve to prior BCMA-directed therapies. Additionally, RRMM pts received prior BCMA-targeted therapy may also benefit from teclistamab. Clinical trials of bsAbs as monotherapy or in combination therapy are currently recruiting pts with RRMM or NDMM.

## Data Availability

The material supporting the conclusion of this study has been included within the article.

## Abbreviations

ASCO: American Society of Clinical Oncology
BCMA: B-cell maturation antigen
bsAbs: Bispecific antibodies
CR: Complete response
CRS: Cytokine release syndrome
GPRC5D: G protein-coupled receptor family C group 5 member D
ICANS: Immune effector cell‐associated neurotoxicity syndrome
FcRH5: Fc receptor-homolog 5
m: Months
mDoR: Median duration of response
mFU: Median follow-up
MM: Multiple myeloma
mPFS: Median PFS
mOS: Median OS
NR: Not reached
ORR: Overall response rate
OS: Overall survival
PFS: Progression-free survival
prior T: Prior T-cell-directed therapies
pts: Patients
QW: Quaque week
Q2W: Quaque 2 weeks
RRMM: Relapsed/refractory MM
TI: Transplant ineligible
*vs*: Versus
